# The use of complementary and alternative medicine by people with cardiovascular disease: a systematic review

**DOI:** 10.1186/1471-2458-12-299

**Published:** 2012-04-26

**Authors:** Suzanne J Grant, Yu Sun Bin, Hosen Kiat, Dennis Hsu-Tung Chang

**Affiliations:** 1 Centre for Complementary Medicine Research, University of Western Sydney, Locked Bag 1797, Penrith South DC, NSW 2751, Australia; 2 Cardiac Health Institute, 173 Shaftsbury Road, Eastwood, NSW 2122, Australia; 3 The Australian School of Advanced Medicine, 2 Technology Place, Macquarie University, Sydney, NSW 2109, Australia

## Abstract

**Background:**

Complementary and alternative medicine (CAM) may offer benefits as well as risks to people with cardiovascular disease. Understanding the prevalence and the nature of CAM use will encourage beneficial CAM therapies, prevent potential herb-drug interactions and foster communication between patients and physicians.

**Methods:**

A systematic search of eight bibliographic databases was conducted for studies that investigated CAM use in patients with cardiovascular diseases. Two independent reviewers selected relevant abstracts and evaluated the quality of included studies.

**Results:**

Twenty-seven studies were included. Prevalence of CAM use in cardiac patients ranged from 4% - 61%. Biologically-based therapies usage ranged from 22% to 68%. Herbal medicines were used by between 2% and 46%. A large proportion of patients did not inform medical practitioners about their CAM use and up to 90% of treating physicians did not discuss CAM use with their patients.

**Conclusions:**

CAM use in patients with cardiovascular disease appears common. The findings suggest that the effects of CAM on medical management of cardiovascular disease may be overlooked and that patient-physician communication need to be strengthened.

## Background

Cardiovascular disease (CVD) is the leading cause of morbidity and mortality in Australia. CVD affects four million Australians and accounted for 38% of all deaths in 2004 [1]. Around 62% of people with CVD take medication for their condition, and given the chronic nature of CVD, this is an important part of long-term management. Effective medical management of CVD may be compromised by inappropriate use of complementary and alternative medicine (CAM). CAM use is estimated at between 9% and 65% globally [2]. In Australia, one in two people regularly use CAM, and consumers spend more money on CAM than prescription drugs [3].

The Australian Therapeutic Goods Administration (TGA) defines complementary medicine as medicines containing herbs, vitamins, minerals, nutritional supplements, homoeopathic medicines and certain aromatherapy products [4]. The TGA’s definition does not cover some important non-ingestible CAM modalities such as osteopathy, massage, reiki, qigong, yoga and meditation. These modalities are included in a more comprehensive definition from the US National Centre for Complementary Medicine and Alternative Medicine (NCCAM) [5].

In cardiac patients, the use of CAM offers both risks and benefits. For instance, physical therapies such as qigong appear helpful for hypertension while coenzyme Q10 supplements have favourable effects in those with heart failure [6,7]. Biological therapies such as dietary supplements and herbal medicine may interfere with the action of prescription medications, a potentially harmful consequence. Herb-drug interactions are of concern in cardiac patients because of the narrow therapeutic window and range of cardiac medications. People with chronic diseases frequently use CAM therapies to manage their condition, and thus increase exposure to herb-drug interactions [8]. It is therefore important to understand the prevalence and the nature of CAM use in this patient cohort to encourage beneficial CAM therapies and to prevent potential herb-drug interactions. In the current study, we conducted a systematic review of the literature to determine the prevalence of CAM use by cardiac patients.

## Methods

### Literature search strategy

We searched eight databases for articles published in peer-reviewed journals that reported primary data on the prevalence of CAM use among people with cardiovascular disease. Details of the search strategy are provided in Table 1. The period of the search was from the inception of each database until 20 April 2010.

**Table 1:** Databases, MeSH keywords, and qualifiers used in the search strategy

**Database** **From inception – 20 April 2010**	**Search terms**
AMED	MeSH: ‘cardiovascular diseases’ [exploded] AND (‘complementary therapies’ [exploded] OR ‘dietary supplements[exploded] OR ‘plants, medicinal’ [exploded] AND ‘data collection’ [exploded] Where MeSH terms were unavailable, the following terms were combined: [complementary medicine$ or complementary therapy$ or alternative medicine$” or “supplement$ or herb$ or homeopath$ or osteopath$ or acupuncture or Chinese medicine$ or mind-body therapy$] AND cardiovascular disease AND [‘utilisation” OR “utilization” OR ‘ prevalence’ OR ‘use’]
Blackwell Synergy	
CINAHL	
Health & Society	
PubMED Entrez (indexes Medline and Biomed Central)	
Scopus (indexes EMBASE)	
Science Direct	
Web of Science	

Studies were included if they reported the prevalence of CAM use in patients either attending for cardiac care or reporting a cardiovascular condition. If abstracts were unavailable or unclear, the full-text article was retrieved. Studies were excluded where the (i) study methods were not described, (ii) data on participants with cardiovascular disease could not be separated from other study populations, or (iii) the study was not in English. Reference lists were manually searched to locate articles not found by the above methods. Two independent reviewers (Grant and Chang) examined titles and abstracts of the articles for inclusion or exclusion. Disagreements were resolved by reviewing the full papers and discussion. Data was extracted according to a predefined format covering the study methodology (Table 2), participant demographics, and CAM use (Table 3).

**Table 2:** Quality Assessment of Studies

**QAT Item**	**Brief Definition**	**Points Awarded**	**Frequency of studies % (n)**	**Reference**
*STUDY METHODS*				
Low risk	Prospective/current data collection	2	11 (3)	[9]‐[11]
Some risk	Retrospective data collection <12 months	1	78 (21)	[12]‐[32]
Piloted questionnaire (or interview schedule)	Any pilot or previous use of study material	1	26 (7)	[13,22,23,25,28,29,31]
Address potential sources of bias	Report efforts to address nonresponsive bias or information bias	1	7 (2)	[11,29]
Adjust for potential confounders	Any adjustment of confounders in analyses of variables associated with CAM use	1	44 (12)	[9,11,12,14,15,22]‐[24,26,32]‐[34]
*SAMPLING*				
Response rate	Where response rate = (no. of participants in the study/No of people invited to take part) x 100	1	48 (13)	[9,11,12,14,15,17]‐[19,21,26,29,32,34]
Representative sampling strategy	Any attempt to a representative sample of the larger population	1	26 (7)	[9,11,12,23]‐[25,30]
*PARTICIPANT’S CHARACTERISTICS*				
Specific diagnosis	Report participants’ diagnoses	1	100 (27)	All studies
Indicator of socioeconomic status	Report participants’ socioeconomic status	0.5	67 (18)	[9,12]‐[16,18,19,22]‐[24,28,30]‐[33,35]
Age	Report participants’ ages	0.5	100 (27)	All studies
Ethnicity	Report participants’ ethnicity	0.5	56 (15)	[10,12,14,16,19,22]‐[24,26,30]‐[35]
Gender	Report participants’ gender	0.5	100 (27)	All studies
*CAM USAGE*				
CAM definition	Information about the definition of CAM/a list of CAM modalities provided to participants	2	74 (20)	[9,11]‐[20,22]‐[25,27,29]‐[33,35]
Use of CAM modalities assessed	Report the prevalence of use of specific CAM modalities	1	100 (27)	All studies
Frequency/duration of CAM uses	Report how often or for what duration the CAM were/are used by study participants	1	19 (5)	[24,25,28,30,32]
Reasons for CAM use	Report the reasons for the use of CAM by study participants	2	56 (15)	[9,12,15]‐[18,22,23,26,28,30,31,34,35]

**Table 3:** Characteristics of studies

**Author (Year)**	**Mode of data collection**	**Sample size**	**Diagnosis**	**CAM Modalities Investigated**	Prevalence* (%)
Ackman et al. (1999)	Standardised, self-administered survey	180	CHF	Vitamins/minerals, nutritional supplements, health food or herbal products; OTC medication	59^
Ai et al. (2004)	Combination face-to-face interviews and telephone survey	225	Cardiac surgery	Relaxation, spiritual healing, herbal medicine, megavitamins, acupuncture, chiropractic, massage, biofeedback, hypnosis, imagery, homeopathy; *includes exercise.*	80.9
Albert et al. (2009)	Structured telephone or face-to-face interview	374	Heart failure	Vitamins, minerals, herbal products, OTC medication	11.5 ^^
Amira & Okubadejo (2007)	Semi-structured interview (face-to-face)	225	Hypertension	NIH Categories: whole medical systems, mind-body therapies; dietary supplements & herbs; energy therapy and manipulation & touch therapy; plus sub-categories.	39.1
Artz et al. (2006)	Combination of standardised survey (face-to-face) and medical records	315	CVD	Non-vitamin/mineral dietary supplement, vitamin/mineral dietary supplement.	4
Barraco et al. (2005)	Semi-structured face-to-face interview	223	CAD	Folk remedies, herbal therapy, homeopathy, megavitamin therapy (not daily vitamin), minerals (not calcium or iron), native American medicine, Tibetan medicine, traditional Chinese medicine *(includes exercise and prayer as CAM)*	63
Blackmer and Jefromova (2002)	Standardised telephone survey	136	Stroke or subarachnoid haemorrhage	Acupuncture, massage, chiropractic, reflexology, magnetic therapy, hyperbaric oxygen, herbal supplements, vitamins, spiritual healing, reiki, chelation, relaxation	26.5
Buettner et al. (2007)	Combination of standardised survey (face-to-face) and medical examination; part of the annual National Health and Nutrition Examination Survey (NHANES)	1066	CAD or Stroke	Vitamins, minerals, other dietary supplements	61
Chagan et al. (2005)	Structured face-to-face interview	198	CVD	Biologically based therapies (herbal medicine, Vitamins & minerals)	42
Dal Corso et al. (2007)	Semi-structured face-to-face interview	153	CHF	Herbal remedies, integrators (vitamins, minerals, salts), OTC medications	30^
Decker et al. (2007)	Combination of face-to-face interview, and medical records.	596	CAD	Biofeedback, acupuncture, relaxation therapy, home remedies, and chelation therapy	19
Gohar et al. (2008)	Standardised, self administered survey	153	Hypertension	NIH Categories: whole medical systems, mind-body therapies; dietary supplements & herbs; energy therapy and manipulation & touch therapy; *(includes prayer)*	37.9
Greenfield et al. (2008)	Standardised, self administered survey;	422	CVD	Open ended question on CAM; vitamins & minerals, exercise, acupuncture, homeopathy, chiropractic, massage	9.2
Krasuski et al. (2006)	Standardised, self administered survey	210	CVD	Herbal medications, vitamin supplements (unless prescribed), dietary supplements, visits to alternative medicine providers	54
Lee and Kim (2009)	Structured face-to-face interviews	1434	Hypertension	Dietary supplements	23.7
Leung et al. (2008)	Standardised, self administered survey (mail out)	661	CAD	Mind-body therapy: meditation, yoga, qigong, tai chi, relaxation techniques, deep breathing exercise, visualisation, guided imagery, quiet sitting, mantra, muscle relaxation, and other (not including prayer)	35.1
Liu et al. (2000)	Standardised, self administered survey	263	Cardiac surgery	Ayurveda, Acupuncture, Biofeedback, Chelation, Chiropractic, Energy healing, guided imagery, Herbs, Homeopathy, Hypnosis, Massage therapy, Meditation/relaxation, Naturopathy, Nutritional therapy, Prayer/pastoral counselling, qi gong, reflexology, tai chi, vitamins, yoga, other	75
Martinez-Selles et al. (2004)	Standardised, self administered survey	65	CHF	CAM use was asked in the context of “use of over the counter drugs and alternative medicine”	12
Pharand et al. (2003)	Structured face-to-face interviews	306	CVD	Vitamins or mineral products, nutritional supplements, health food or herbal products (home remedies, oriental remedies)	22.9^
Quan et al. (2001)	Standardised, self administered survey	5854	CAD	Chelation therapy	7.9
Shafiq et al. (2003)	Structured structured face-to-face interviews	521	Hypertension	Ayurvedic medicine, herbal medicine, homeopathy, spiritual healing, diet therapy and acupuncture	63.9
Stys et al. (2004)	Physician interview	187	CVD	Vitamin, mineral or herbal supplements	57
Wong et al. (2003)	Physician interview	107	CVD	Herbal medicine	26
Wood et al. (2003)	Combination of structured phone interview, medical records as part of a larger study: Improving Cardiovascular Health in Nova Scotia (ICONS)	107	CVD	Megavitamins, Herbal therapy, Other nutritional supplements, Chiropractic, Massage therapy, Acupuncture, Homeopathy, Folk remedies, Exercise, Energy healing, Chelation,Biofeedback, Relaxation therapy, Spiritual healing, Hypnosis, Self-help groups *(includes exercise)*	64
*Yeh et al. (2006)	Structured face-to-face interview; 2002 National Health Interview Survey	10572	CVD	NIH Categories: whole medical systems, mind-body therapies; dietary supplements & herbs; energy therapy and manipulation & touch therapy; *(excluding prayer)*	36
Yilmaz et al. (2007)	Semi-structured face-to-face interview	310	CVD	Herbal medicine	26.5
Zick et al. (2005)	Standardised, self administered survey	252	CHF	Herbs, vitamins, minerals, amino acids, other	32.5

* Refers to the prevalence reported in the study of the CAM modalities investigated.

^ No overall prevalence reported to the CAM, the figure presented here is for dictary supplement usage.

^^ No overall prevalence reported for CAM, the figures presented here refer to multivitamin use and herbal therapy use respectively.

### Quality assessment

There is a paucity of methods for assessing the quality of observational studies [36,37]. Bishop et al. (2010) recently developed a Quality Assessment Tool (QAT) for a systematic review of the prevalence of complementary medicine use in paediatric cancer based on the principles of the STROBE statement [38]. We used a modified version of the QAT in the present review (Table 2). Like Bishop et al. (2010), we weighted these items according to their respective significance through consensus of the authors [38] and the two independent reviewers assessed each study using the QAT, with disagreements resolved by discussion.

### Statistical analyses

There are two methods commonly used to summarise prevalence rates across observational studies: variations in frequency estimates can be displayed graphically or summarized with quantiles around measures of central tendency [39].We chose to display the results graphically with no meta-analysis due to the variation in definitions of CAM and the heterogeneous methodologies of the included studies.

## Results

Our database search found 2,124 articles, of which 112 full-text articles were retrieved. Three studies were identified that utilised the same dataset of the 2002 USA National Health Interview Survey [12,40,41]. We included only Yeh et al. [12] which covered the cohorts of the other two studies. In total the search identified 27 studies suitable for inclusion in our review (Table 3). All were cross-sectional studies.

### Study characteristics

#### Scope

The bulk of the studies looked at CAM use in general. Three of these utilised the National Institute of Health’s defined categories of CAM [12]‐[14], while eight studies provided comprehensive yet differing definitions of CAM [9,15]‐[20,33]. Two studies did not define CAM but used open-ended questions to elicit the CAM therapies utilised [21,34]. Thirteen studies looked specifically at biological-based therapies, such as vitamins, minerals and dietary supplements [10,22]‐[26], herbal medicine only [27,28] or a combination of both [29]‐[31,35] and one study reported on the use of chelation therapy following angioplasty [11]. The remaining study looked specifically at mind-body therapy [32].

Most studies reported on CAM prevalence in a cohort of people with cardiovascular disease although some reported on a specific diagnosis. Two studies reported on CAM use in people with stroke [17,23], five on chronic heart failure [21,26,29,30,35] and four examined CAM prevalence in people with hypertension only [13,14,20,24].

#### Location/setting

Twelve of the 27 studies were undertaken in the United States [10,12,15,16,18,19,22,23,26,30,31,33] and six in Canada [9,11,17,25,29,32]. The remaining nine were conducted in the UK [14,34], Hong Kong [27], India [20], Italy [35], Korea [24], Nigeria [13], Spain [21], and Turkey [28].

Participants were typically recruited in outpatient clinics or hospital settings. Six of the 27 studies used national survey data [9,11,12,22]‐[24].

#### Sample size

Sample sizes ranged from 65 [21] to 10572 [12]. The two largest studies were the US National Health Interview Survey [12], and a survey of chelation in 5,854 coronary angiography patients in Canada [11].

#### Data collection

In these studies, research methods used for data collection included standardised, self administered surveys [11,14,18,19,21,26,29,32,34] and face-to-face interviews using a structured [12,24,25,31] or semi-structured instrument [10,13,16,20,27,28,35]. The remaining studies used mixed methods, either a combination of face-to-face and phone interview [15,30], face-to-face interview and a medical examination [23], data collection from medical records, [22,33] or a phone interview and information from medical records [9]. One study used phone interview alone [17].

#### Study quality

The quality of the studies examined in the present review varied significantly ranging from meeting only 25% of the QAT criteria [21] to meeting more than 75% of the QAT criteria [12]. Eight studies, representing 30% of the total, scored less than 50% on the QAT (QAT ≤ 8/16) [10,13,19,21,26,27,33]‐[35]. Most studies clearly reported the CAM modalities that were being assessed and provided a reasonable definition although the lists of CAM modalities included in these definitions varied considerably. The included studies were fairly diligent in their collection of demographics (age, gender, ethnicity and socioeconomic data) although some studies conducted in regions or countries of more homogenous ethnicity did not report ethnicity of the study population. Data collection was commonly retrospective and the studies were generally poor in their reporting of the frequency and duration of CAM use, validation of the survey instrument, and providing a representative sample.

### Prevalence of CAM use among CVD patients

The prevalence of CAM use among people with CVD ranged from 4% [22] to 61% [23]. CAM modalities investigated were in some studies limited to types of biological-based therapies [10,21]‐[24,26]‐[29,31], a single therapy such as mind-body therapy [32] or chelation [11]. In the seven studies which used comprehensive lists or definitions of CAM [12]‐[14,17,18,20,33], prevalence of CAM usage ranged from 19% to 64%.

**Figure 1:**
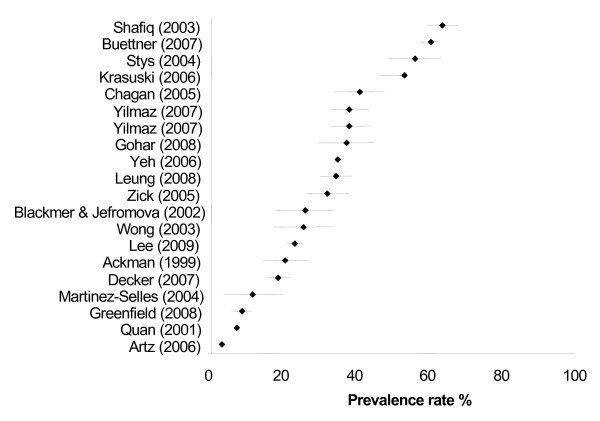
Prevalence (% ± 95% confidence interval) of CAM usage by people with CVDs.

Six studies were excluded from Figure 1 as we could not separate the use of exercise [9,15] and/or prayer [13,16] from the overall CAM usage data. In the other three studies [25,30,35], OTC medications or nutritional supplements and health food could not be extracted from the overall CAM figure.

### Types of CAM modalities used

*Biological based therapies* Of the 23 studies that reported on biologically-based therapies, only three provided an overall prevalence figure and breakdown into herbal medicine or dietary supplements [10,12,20]. Seven studies [10,12,13,18,23,26,31] provided data on overall biologically-based therapies usage that ranged from 22% [12] to 68% [18]. Eight studies provided only separate figures for herbal and/or dietary supplements and did not ascertain an overall prevalence. Biologically-based therapies were reported as an average of two supplements per user [28,31] while another study recorded an average of three supplements per user [10].

**Figure 2:**
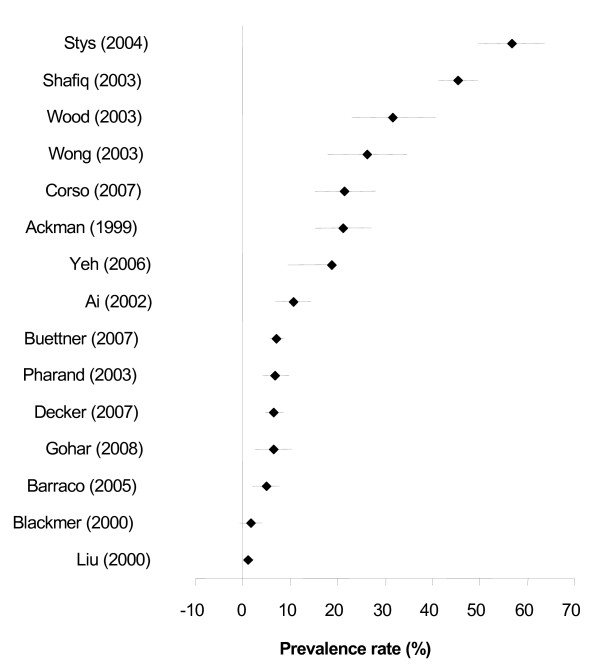
Prevalence (% ± 95% confidence interval) of herbal medicine use in people with CVDs.

*Herbal Medicine* In sixteen studies reporting the prevalence of herbal medicine use by people with CVD, herbal medicines were used by between 2% [17] and 46% [20] of respondents (Figure 2). Data for Zick (2005) was not presented in Figure 2 as herbal medicine could not be separated from vitamins and minerals. The top five individual herbs utilised were echinacea (2% [26] to 14% [30]), garlic (1% [28] to 69% [13]), ginger (1% [29] to 24% [13]), ginkgo (1% [26] to 9% [30]), and ginseng (1% [25] to 6% [29]).

**Figure 3:**
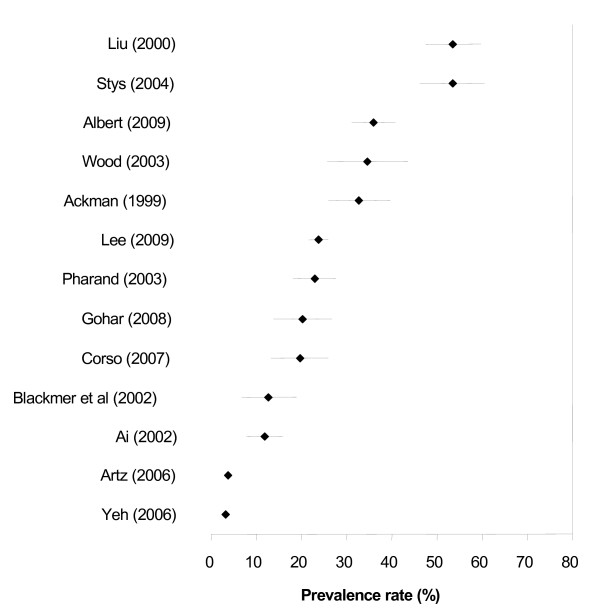
Prevalence (% ± 95% confidence interval) of Vitamin and Mineral Use in People with CVDs.

*Vitamin, Minerals and Other Dietary Supplements* The thirteen studies that provided prevalence data on vitamins, minerals and other dietary supplements showed usage to be between 3% and 54% (Figure 3). The most common supplements were vitamin B/B12 or B complex, vitamin C, vitamin E, calcium, glucosamine/chondroitin, coenzyme Q10, calcium and magnesium.

*Mind-body therapies* Seven studies [12,14,15,17,19,32,33] reported on the prevalence of mind-body therapy (MBT) usage, which ranged from 2 to 57%. An additional study [13], reported on the prevalence of mind-body connection (3.4%) but this was limited to prayer and fasting. Deep breathing and meditation were the most common MBT therapies among the CVD participants. The main reasons for MBT usage were for psychological and emotional well-being.

### Physician awareness of patients’ CAM use

Physician awareness of their patient’s CAM therapy use was reported in six studies [12,16]‐[18,28,31]. Awareness was lowest in the Turkish study, 8% [28], and was between 39-65% in the others [12,16,18,31]. The reason for non-disclosure to physicians about CAM use was investigated in two studies. Reasons given were fear that their physician might disapprove [17] and that their physician had not enquired about their CAM use [16].

### Reasons for CAM use

Twelve studies investigated CAM users’ perceptions of CAM or reasons for CAM use [9,12,15]‐[18,20,26,30,31,34,35]. CAM was thought to be of greater benefit than conventional medications by 15% of users in two studies [9,18]. One study reported that 59% of patients chose CAM due to adverse drug reactions to conventional therapy [20]. Overall well-being and promoting good health were the main reasons given for taking vitamins and herbal therapy in one study [30]. Two studies reported that CAM was a perceived remedy by 40% [15] and 57% [9] of users, and 28% reported that it had a proven benefit for their condition [9].

The use of CAM specifically to treat cardiovascular disease was reported in six studies [9,16,18,26,28,31]. In patients with heart failure, up to 82% of CAM users were taking a supplement specifically for cardiovascular health [26]. Wood et al. (2003) and Chagan et al. (2005) reported that 43% [9] and 65% [31] of CAM users used CAM for their CVD, and in the remaining four studies, between 7% [18] and 23% [16,28] had used CAM for their CVDs.

Five studies reported that users of CAM felt that biologically-based therapies were safe or had fewer side effects than conventional medications [9,15,18,31,35]. This perception ranged from 29% to 45% of CAM users in four of the five studies, and in the remaining study 3% of patients reported that the reason for using CAM therapies was due to fewer side effects compared to conventional treatment [9].

### Concurrent use of biological-based therapies and prescription medication

Seven studies reported data on the concomitant use of prescription medication and biological- based therapies [9,15,18,22,23,27,31]. Aggregation of data was difficult due to variations in method although useful insights are provided by these investigations. On average, cardiovascular patients consumed seven prescribed medications and two herbal, vitamin or mineral products daily [9]. One study undertook an examination of medical records and identified 42 potential herb-drug interactions [31]. Two studies found that people with cardiovascular disease had taken dietary supplements or herbal medicine with warfarin [9,27]. In the Canadian study, 13% of patients had used a dietary supplement in conjunction with warfarin, amiodarone, sotalol, or digoxin [9]. In the Hong Kong study, 26% of patients on warfarin had taken a herbal medicine in the week prior to interview [27]. Taking medication such as aspirin, beta-blockers, ACE inhibitors, statins and warfarin did not appear to alter the likelihood of using biological-based therapies [18].

Compliance to prescribed medication was examined in three studies [14,35,42]. The use of biological-based therapies was found to not effect compliance to antihypertensive medication, except in one study where females had lower compliance [14]. In a study of outpatients with heart failure taking herbal medicine reported that 1 in 22 patients reduced or interrupted heart failure medications [35].

## Discussion

The results indicate that CAM use is common in cardiac patients but the prevalence of CAM use varies significantly (4%-61%). The large range found is consistent with other reviews in the CAM literature [2,43]. The findings have strong implications for both clinical practice and research in this field. The lack of specific and consistent definitions of CAM in the reviewed studies contribute to the variability observed and has made generalisations difficult. A standard and complete definition of CAM use would facilitate meta-analysis in future studies [43].

The reasons for CAM use vary remarkably between different cohorts although six studies did find that patients used CAM specifically for the management of their CVDs. Although our review has found inconsistent results with regard to the association between CAM use and compliance, some evidence exists to suggest that CAM use may affect the compliance of prescription medicines [14,35]. A patients’ belief that their CAM usage help manage their CVD may give them a false sense of security that may lead to a reduction of their concurrently medicines.

We found across the studies that patients with CVD are likely to be using more than one CAM product simultaneously [9,10,23,28,31]. There appeared to be little awareness that there may be interactions with their prescription medication, having potential additive or negative effects on the therapeutic levels of their medication or a harmful adverse effect [28,31,35]. Supplemental potassium was taken by 20% of patients in one study [29], which can result in adverse outcomes when used together with commonly prescribed medications such as angiotensin converting enzyme inhibitors, aldosterone receptor antagonists, or angiotensin receptor blockers. The potential effects and implications of herb-drug interaction with CVD medications have been discussed in detail elsewhere [44,45].

It is of great concern that a large proportion of medical practitioners (35 ‐ 92%) were unaware of the CAM use by their patients [12,16]‐[18,28,31]. Although patients may be reluctant to disclose CAM use because they fear disapproval, doctors also do not appear to be asking their patients about CAM use [31].

The results of our review are restricted in several aspects. For example, the quality of the reviewed studies was highly variable. Data collection in most of the studies was retrospective. This led to high variability in the resulting prevalence rates and may have introduced significant recall bias. Many studies also lacked the use of a validated survey, a representative sample, and rarely did the study authors address potential biases arising from their methods. Many of the studies failed to report the frequency and duration of CAM use. Several studies made use of clinical samples, but few provided objective data on the health status of the patients. The results presented here should therefore be treated with caution. We were only able to include studies published in the English language and therefore the findings have limited generalisability outside of English-speaking regions. However we note that it is within countries such as Australia and the United States where use and concerns about CAM are growing the fastest. Therefore the current study provides much needed information on these areas.

## Conclusions

The use of CAM is common in patients with cardiovascular conditions. A high proportion of patients using CAM believe CAM has remedial benefits, and were at least as safe as or safer than their prescribed treatments. CVD patients were often unwilling to inform their medical practitioners of CAM use. Many commonly used CAM products have the potential to interfere with the intended action of prescription medications. In addition, the CAM use may have negative impacts on the compliance with prescription medications. Better education of patients, medical practitioners and pharmacists is needed to improve understanding of the risks and benefits of CAM use. Future studies are required to determine the impacts of CAM use in CVD patients, particularly its clinical and prognostic impact when used in conjunction with prescription medicines.

## Competing interests

The authors declare that they have no competing interests.

## Authors’ contributions

SJG, YSB and DHC conducted the literature review and evaluated the articles recruited in the study. HK contributed to the design of the study. SJG drafted the manuscript and all other authors contributed to its preparation. All authors read and approved the final manuscript.

## Pre-publication history

The pre-publication history for this paper can be accessed here:

http://www.biomedcentral.com/1471-2458/12/299/prepub
